# Personalized medicine – a tailored health care system: challenges and opportunities

**DOI:** 10.3325/cmj.2012.53.211

**Published:** 2012-06

**Authors:** Soulla Louca

**Affiliations:** Department of Management & MIS, University of Nicosia, Nicosia, Cyprus

## Abstract

**Abstract:**

The vision of the future health care should be a system in which patient care is consistently improved through the use of information on the individual patient’s genomes and their downstream products. This requires the exploration of strategic relationships among various disciplines such as life sciences, mathematics, physics, chemistry, and information and communication technology, and constellation thinking to propose new ways for the diagnosis and therapy of diseases, integrated with a planned trans-disciplinary scientific approach involving all interested parties. Connecting high-quality trans-disciplinary scientists on a pan-European level through programs such as the Cooperation in Science and Technology (COST) can support capacity building and increase the impact of personalized medicine research on regulatory bodies, decision makers, pharmaceutical and insurance companies, and the paying public. Such group effort could enable breakthrough scientific developments leading to new concepts and products and thereby contributing to the strengthening of Europe’s research and innovation capacity while reforming the health care system.

Basic science on the genetic background of individual receptiveness toward drugs has progressed over the years. Differences in metabolic capacity have been for example in the focus of the first the Cooperation in Science and Technology action in biomedicine (COST B1, 1986). Since then, progress has been made in the research on the pharmacodynamic aspect to clarify individual vulnerability. Lately, additional progress has been made owing to innovative tools for diagnosis at the molecular level, leading steadily into a transformation of the health care system; a transformation fueled by the adoption and the rapid developments of information and communication technology (ICT), genomics and related disciplines, as well as the cultural drivers of personalized medicine (1). The virtual patient model enabled by personalized ICT services and providing genetic and genomic information of every individual based on a laboratory-on-chip technology and bio-nanotechnology promises personalized medication and genome profiles and other “-omics” of individuals (2). Personalized medical care is designed to get the individual patient a drug that will be therapeutically active while minimizing the adverse effects.

Personalized medicine encompasses not only tailor-made drugs at the correct dose for the right patient, but also incorporates management of our personal data and clinical information (3). Realizing the potential of personalized medicine requires new methods for processing of the deluge of genomic data and translation of the findings into medical practice (2). Biology is being captured in software and hardware through the modeling of genes, proteins, cells, and human organs. This theoretical abstraction of biology into accurate models involves the disciplines of mathematics, physics, and chemistry, while data gathering, simulation, and visualization are involving all aspects of ICT. The upheaval in the life sciences enabled by ICT requires new computing capabilities, sophisticated algorithms, a vast range of software products, internet technologies, as well as advanced data management capabilities for the large torrent of data. The creation of the virtual patient model, a personal simulation of the human body becomes mandatory for a faster, reliable, and successful health care system. The development of affordable next-generation high-throughput technologies allows generation of data from the entire genome, transcriptome, epigenome, etc, from a single (routine) clinical specimen (4,5). These technologies are expected to influence the fundamentals of the current practice of “reactive” medicine to a more systemic, structured, and evidence-based approach, to change the current classification/definition of disease entities and to influence to a great extent the therapeutic protocols. As a consequence, ICT is a fundamental part of the process of understanding the human body and life in itself as a complex biological system, speeding-up the whole drug discovery/development process, providing new targets for selective inhibitors, and reducing costs.

The appropriate use of ICTs is probably the most important strategy to translate information from “-omics” research into clinically relevant products and technologies and revolutionize life sciences.

## Challenges and opportunities

The numerous challenges faced by scientists slow the progress in personalized medicine, subsequently delaying the advantages and the opportunities for the patients.

Identifying each individual's reaction for absolute personalized medicine is neither easy and straightforward from a research perspective nor practical from a pharmaceutical, diagnostic, or prognostic perspective. Stratification of the patients’ responses within groups might be more practical and manageable as a first step to personalized medicine before we are technologically and scientifically advanced to apply personalized medication. Several initiatives in Europe including the UK’s Stratified Medicine Innovation Platform (6), Sweden’s Biobank Program (7), BIOMEDREG in Czech Republic (8), and the Munich Biotech Cluster are already working toward this goal (9). The US Food and Drug Administration is in the process of evaluating medical products and integrating the various medical product regulatory authorities provided by Congress in the Federal Food, Drug and Cosmetic Act to develop effective mechanisms for successful implementation of personalized medicine in the USA (10).

In addition to the scientific challenges faced by scientists, several other issues have to be addressed for a triumphant implementation of this new health care system brought upon us by personalized medicine.

Well trained and educated personnel are required to design, maintain, and use these environments, which employ a whole new approach for the drug development process, providing new and validated drug targets for the development of drugs with a much higher therapeutic success rate, safety, and further cost efficiency (11). The education of medical practitioners and the public as a whole on these new developments remains a formidable challenge.

We are empowering the patients with predictive and preemptive information to manage their own health. They will be able to harness the knowledge to keep on being healthy or take advantage of treatments tailored to their genetic profile. On the other hand, these predictive abilities might make one decide that this is not a welcome method as individuals will have the power to know ahead of time whether they are susceptible to certain diseases based on their genetic profile (12). And what about privacy issues? Parents can decide at an early age that their child is to have a genetic profile. As an adult, that child might not want to know what awaits him/her in the future. This raises another question – how do we interpret prognostic information and what do we actually want to predict and where to intervene? Especially for children, a wrong prognosis/interpretation will generate serious problems that will follow him/her for the rest of his/her life.

Is this foretelling information going to be the privilege of the few? Are we going to end up with a health care system that will not be accessible to everybody? Will the patient gain from the new approach when clinically beneficial new products and procedures are translated into affordable clinical practice? The policy challenges, including encouraging and financing innovation related to the field, need to be surmounted for effective individually tailored medical interventions, simultaneously reforming the health care system.

Stratified medicine and the potential use of biomarkers are foreseen to have a major effect on both clinical practice and the development of new drugs and diagnostics (11-14). The approach of therapies being matched with specific patient population characteristics uses clinical biomarkers. This transforms product development strategies and market structures, having a strategic and economic impact on the whole health care system. Addressing the salient issues facing personalized medicine underpins the regulatory framework that governs it and the markets that drive its development and adoption. The major obstacles for making personalized medicine available to patients requires basic, translational, and also regulatory science, especially in the areas of ethical, legal, and social issues (3). The vision should be a health care system in which patient care is consistently improved through the use of information on the individual patient’s genomes and their downstream products (ie, transcriptomes, proteomes, and metabolomes). This will require the exploration of strategic relationships among all interested parties including researchers, clinicians, policy makers, and pharmaceutical and insurance companies. Constellation thinking will offer new ways for the diagnosis and therapy of diseases, integrated with planned trans-disciplinary scientific approaches, involving various disciplines, such as life sciences, mathematics, physics, chemistry, and ICT. Through programs such as COST, high-quality trans-disciplinary scientists should be connected to support capacity building, and research on regulatory bodies, decision makers, and pharmaceutical and insurance companies. Only interdisciplinary activities will enable breakthrough scientific developments leading to new concepts and products and thereby contributing to strengthen Europe’s research and innovation capacities while simultaneously reforming the health care system.

Personalized medicine should not be a promise for the future, but a common scientific and technological effort offering patients and health care practitioners up-to-date diagnostics and therapeutics.
